# Cost-utility analysis for sublingual versus intravenous edaravone in the treatment of amyotrophic lateral sclerosis

**DOI:** 10.1186/s13023-024-03381-w

**Published:** 2024-10-28

**Authors:** Chang Liu, Yao Wu, Fangxu Wang, Shuang Sun, Jiayin Wei, Libo Tao

**Affiliations:** Center for Health Policy and Technology Evaluation, Peking University Health Science Center, Beijing, 100191 China

**Keywords:** Edaravone, Sublingual tablet, Amyotrophic lateral sclerosis, Cost-utility analysis

## Abstract

**Background:**

Edaravone has been widely used in amyotrophic lateral sclerosis (ALS) treatment, and a sublingual (SL) tablet has been developed to offer a more convenient alternative for injection. We present a cost-utility analysis to comprehensively evaluate the costs and health outcomes of oral and intravenous edaravone for the treatment of ALS in Chinese medical context.

**Methods:**

Cost-utility analysis of SL tablets of edaravone versus intravenous edaravone at home was performed by constructing a 20-year Markov model of ALS stage 1–4 and death. The data were extracted from the literature with model assumptions. Typical sensitivity analysis and scenario analysis for administering SL tablets at home versus intravenous tablets at the hospital were performed.

**Results:**

In the base case analysis, with SL tablets and intravenous injections both at home, the model estimated an additional cost of ¥12,670.04 and an additional 0.034 QALYs over 20 years (life time) of modeling analysis, and the ICER was ¥372,648.24 per QALY. However, in the scenario of intravenous administration at the hospital, SL tablet was demonstrated dominance to intravenous injection.

**Conclusions:**

Using 3 times the GDP per capita of China in 2023 as the threshold, the SL tablet edaravone was not cost-effective in the context of home treatment for both formulationst, but was dominance to intravenous injection in hospital treatment. The results highlighted the importance of treatment context for health economic analysis.

## Introduction

Amyotrophic lateral sclerosis (ALS) is a fatal neurodegenerative disease characterized by progressive degeneration of upper and lower motor neurons, leading to gradual worsening of muscle weakness, muscle atrophy, pyramidal signs, dysarthria, and language impairments, ultimately resulting in death due to respiratory failure [1–3]. The etiology and pathogenesis of ALS remain unclear, and its onset is insidious. The prevalence and incidence of ALS in China were 2.97 per 100,000 person-years and 1.62, respectively [4]. The majority of patients develop the disease after middle age and mostly die within 3–5 years due to respiratory muscle paralysis and pulmonary infections [4, 5]. The financial burden of ALS is huge and increased with longer hours of care and poorer quality of life. An ALS disease burden investigation of southwest China reported 36% of the caregivers’ work were affected, 62% of ALS patients' medical expenses exceeded their family annual income [6]. In summary, ALS causes severe disability and has a high mortality rate, not only impacting the patient's lifespan and quality of life but also placing a heavy economic and care burden on their families and society [7, 8].

Edaravone, a free-radical scavenger, possesses favorable properties for penetrating the brain. It has gained approval for treating acute ischemic stroke and ALS. While edaravone injection has been widely used in ALS treatment, a sublingual (SL) tablet has been developed to offer a more convenient alternative, which was launched in China in September 2022. This SL dosage is suitable for long-term independent administration by ALS patients due to its ease of use. It is accessible to a wide range of ALS patients, including those with mobility impairments who are unable to reach the hospital for intravenous (IV) treatment.

ALS has a significant impact on the lives of patients, but it does not affect their intelligence. With timely and effective intervention and treatment, the progression of the disease can be slowed, and life expectancy can be extended. This allows patients to engage in cognitive work, highlighting the social significance of ALS treatment. The emergence of new dosage forms provides more options in clinical settings. Clinical research indicates that the SL tablet is bioequivalent to the injectable formulation according to the 3-compartment linear disposition model describing the PK profile of edaravone after either IV infusion or SL administration [9, 10]. Currently, the unit price of SL tablets is higher in China, but one study [11] discussed the differences in direct nonmedical costs and indirect costs between oral and IV formulations of edaravone, suggesting that the oral formulation may result in greater cost savings such as diminishing travel costs and wages lost due to travel and appointment time saving. Additionally, the suitability of different administration routes varies, as studies [12–15] have shown that patients perceive differences in health utility values between oral and IV formulations of the same active ingredient drug. Currently, there is no comprehensive evaluation or systematic discussion of the costs and health outcomes of oral and IV edaravone for the treatment of ALS. In this study, we conducted a cost-utility analysis to comprehensively evaluate and discuss the costs and health outcomes of oral and IV edaravone for the treatment of ALS in Chinese medical situation.

## Materials and methods

### Study design

Based on previous research findings, this study explored the differences in cost and health outcomes between the SL and IV formulations of edaravone for treating ALS. A Markov model was developed to systematically evaluate the economic aspects of ALS treatments using both formulations from a societal perspective. Patients in the SL group were assumed to receive SL edaravone (30 mg per tablet), while patients in the IV group were assumed to receive IV edaravone (30 mg per vial). Both formulations have equivalent edaravone content and treatment duration: a total of 28 days, including a treatment period and a drug-free period. The initial course involved 14 consecutive days of administration followed by a 14-day drug-free period. Subsequent courses and beyond consisted of 10 days of administration within a 14-day period, followed by another 14-day drug-free period. The daily dosage is 60 mg (two SL tablets or two vials of IV) per day.

### Model structure

**Fig. 1:**
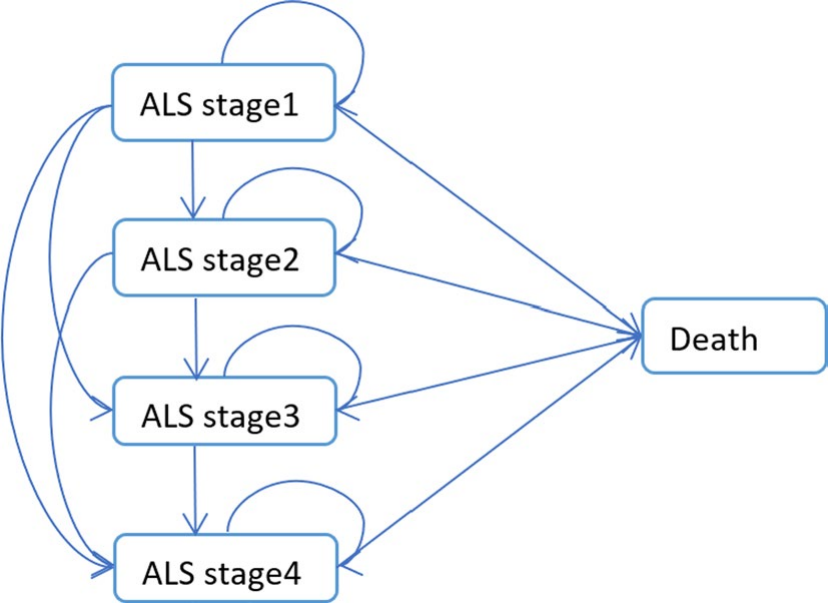
ALS Markov Model. *ALS* Amyotrophic lateral sclerosis

The ALS model was extracted from the Pharmacoeconomic Review Report of Edaravone for the Treatment of ALS [16], released by Canada's Drug and Health Technology Agency, which is the classic model fully demonstrate ALS progress path. Health states included ALS stages 1 to 4 and death, and ALS progression from stages 1 to 4 was irreversible, as depicted in Fig. 1. Health states in the model were defined based on the King’s ALS clinical staging system with five health states: stage 1, functional involvement of one central nervous system (CNS) region; stage 2, functional involvement of two CNS regions; stage 3, functional involvement of three CNS regions; stage 4, functional involvement of between one and three CNS regions plus the need for gastrostomy or non-invasive ventilation (tracheostomy). According to the study of incidence and prevalence of ALS in urban China [4], the average age of ALS in China is 54 years-old, and therefore the starting age of patients entering the model was set as 54 years-old. To capture the long-term outcome of each formulation, a Markov model with a 3-month cycle length and a 20-year time horizon (lifetime) was applied.

As the bioequivalence between SL and IV administration of edaravone for ALS treatment has been demonstrated, we assumed that both formulations have an equal probability of disease progression throughout the entire treatment duration. Table 1 shows the details of the transition probabilities, which were obtained from the aforementioned report, estimated based on clinical data from Pooled Resource Open-Access ALS Clinical Trials database and Thakore’s estimation [17].

**Table 1:** Transition probabilities

From					
To	Stage 1	Stage 2	Stage 3	Stage 4	Death
Stage 1	0.677	0	0	0	0
Stage 2	0.217	0.706	0	0	0
Stage 3	0.064	0.213	0.830	0	0
Stage 4	0.038	0.063	0.118	0.820	0
Death	0.004	0.018	0.052	0.180	1

### Costs

The cost analysis included direct medical costs (medication costs, hospital operating costs, and costs of adverse events), direct nonmedical costs, and indirect costs. medication costs were calculated based on the assumption of both groups of ALS patients adhered to the prescribed drug instructions. The incidence of adverse events was based on the instructions provided for the two drugs. Indirect costs were related to work delay expenses, which were calculated by multiplying the delay time by the per capita disposable income per hour. All cost parameters were sourced from the 2023 database or publicly available government service price lists.

The detailed medical costs are shown in Tables 2 and 3. The parameters related to indirect costs were obtained from the economic evaluation study of oral edaravon. The employment rate for ALS patients was approximately 15%, and the average time lost per hospital visit was 6.23 h [11]. According to the statistical yearbook [18], the per capita disposable annual income in China was ￥36,883 in 2022, from which we estimated the average hourly wage for Chinese residents as ￥18.44. Therefore, the average productivity loss per hospital visit for ALS patients receiving the IV formulation was estimated to be 15% * 6.23 * ￥18.44. Other treatment related costs were assumed to be the equal among two groups and therefore did not involved in the calculation.

**Table 2:** Medical costs

Drug	Price	Daily costs (￥)	Other costs	Treatment course costs (￥)
SL Edaravone	130.00￥/tablet (30 mg)	260.00		2600.00 (3640.00 for the first course)
IV Edaravone	86.83￥/vial (30 mg)	173.66	PICC catheter insertion ¥1847.96 (replaced once per year) PICC maintenance cost ¥103.17 (twice per treatment course)	2085.05 (2779.73 for the first course)

**Table 3:** Adverse event incidence rate and treatment costs per treatment course, 3 months

Adverse events	Rates		Costs (￥)
	SL (%)	IV (%)	
Erythra	0.05	0.70	20.00
Liver damage	0.24	2.81	1985.50
Kidney damage	0.28	0.00	613.00

### Health outcomes

We assumed that the primary difference in effectiveness between the two groups is health utility values. The SL tablet formulation offers advantages in terms of the convenience of taking and eliminating the waiting time associated with IV infusion; thus, patients receiving SL tablets will experience improvements in health utility across health states of ALS stages 1 to 4. Several studies about health utilities of ALS patients was under consideration [19–21]. The health utility values after IV treatment were obtained from Kiebert’s study [19], which focused on utilities of different ALS stages while the other studies grouped ALS patients with different rules or perspectives. Those values were also cited by Tavakoli in his pharmacoeconomic study [22]. The coefficient for the utility improvements with SL tablet treatment was based on Mark's research [15], which discussed the differences in utility values between oral and IV antibiotic treatment for early-stage Lyme arthritis patients.

Health state utility values are shown in Table 4. The improvement in the utility of the SL tablet formulation was evaluated according to Mark's study [15]. The utility adjustment coefficient for patients receiving different delivery methods was set with an adjustment factor of 0.99 for oral treatment and 0.97 for IV treatment. In this study, the SL utility adjustment coefficient between SL and IV was 1.021.

**Table 4:** ALS health state utility

Health states	Utility
ALS stage1	0.740
ALS stage2	0.630
ALS stage3	0.510
ALS stage4	0.370
SL Utility adjustment coefficient	1.021

### Cost-utility analysis

A cost-utility analysis was carried out to compare SL to IV edaravone over a 20-year time horizon with a half-cycle correction applied during calculation. In accordance with Chinese guidelines, a discount rate of 5% was applied to both costs and utilities, with 0–8% considered in the sensitivity analysis. The incremental cost-effectiveness ratio (ICER) was calculated by dividing the difference in mean costs between the two strategies by the difference in mean quality of life years (QALYs) gained. This ICER represents the extra cost that must be spent to gain an additional QALY with the SL formulation.

### Sensitivity analysis

To assess the uncertainty of the results, one-way sensitivity analysis were undertook. Based on health economics evaluation sensitivity analysis methods, A one-way sensitivity analysis was conducted by adjusting cost parameters by ± 20% based on common practices of pharmacoeconomic sensitivity analysis, while effect parameters (transition probabilities, health utility values) were adjusted by ± 10%. For parameters ranging from 0 to 1, values exceeding 1 were set to 1 for calculation purposes. Results from those analyses are visually in a Tornado diagram, sequentially highlighting variables with the most significant impact on the cost-effectiveness outcomes.

A Probabilistic sensitivity analysis (PSA) was carried out using Monte Carlo simulation and calculated new ICERs in 5000 random resamples. The distributions of each variable were set based on previous research experience. Gamma distributions were assumed for cost data and beta distributions for probabilities and utilities. To measure the uncertainty around ICER, 95% CIs around the estimates of incremental cost and incremental effectiveness were calculated using patient level data from the study. Scatter plots were constructed and used to construct a cost-utility acceptability curve, showing the probability that SL edaravone would be cost-effective for different levels of willingness to pay by the decision maker.

### Scenario analysis

The utility adjustment coefficient between SL and IV edaravone was calculated for patients with similar treatment conditions (both treated at home). Under real-world conditions, SL edaravone could be administered at home, while IV edaravone is usually administered in hospitals. Because ALS patients have difficulty moving, treatment conditions always have a great impact. In the scenario analysis, the utility difference of SL edaravone administered at home and IV edaravone administered in the hospital and the transportation cost of the IV group were considered.

## Results

### Cost-utility analysis at the lifetime

This study assumed that 1000 patients with stage 1 ALS enter the SL and IV edaravone groups with equal probability. The evaluation model ran for a 20-year period. In the long term, 82% of patients had a survival period ranging from 3 to 5 years, which is consistent with clinical disease characteristics. 99.9% of patients reached endpoint after 20 years modeling. Compared to IV edaravone, the model estimated an additional cost of ¥12,670.04, while it gained an additional 0.034 QALYs over 20 years in the SL group. The ICER was calculated as ¥372,648.24 per QALY. The baseline analysis results are provided in Table 5.

**Table 5:** Cost-utility results of SL versus IV edaravone

Groups	Costs	QALYs	Incremental cost	Incremental QALYs	ICER (￥/QALY)
SL	￥92,864.81	1.629	￥12,670.04	0.034	372,648.24
IV	￥80,194.77	1.595			

### Sensitivity analysis

**Fig. 2:**
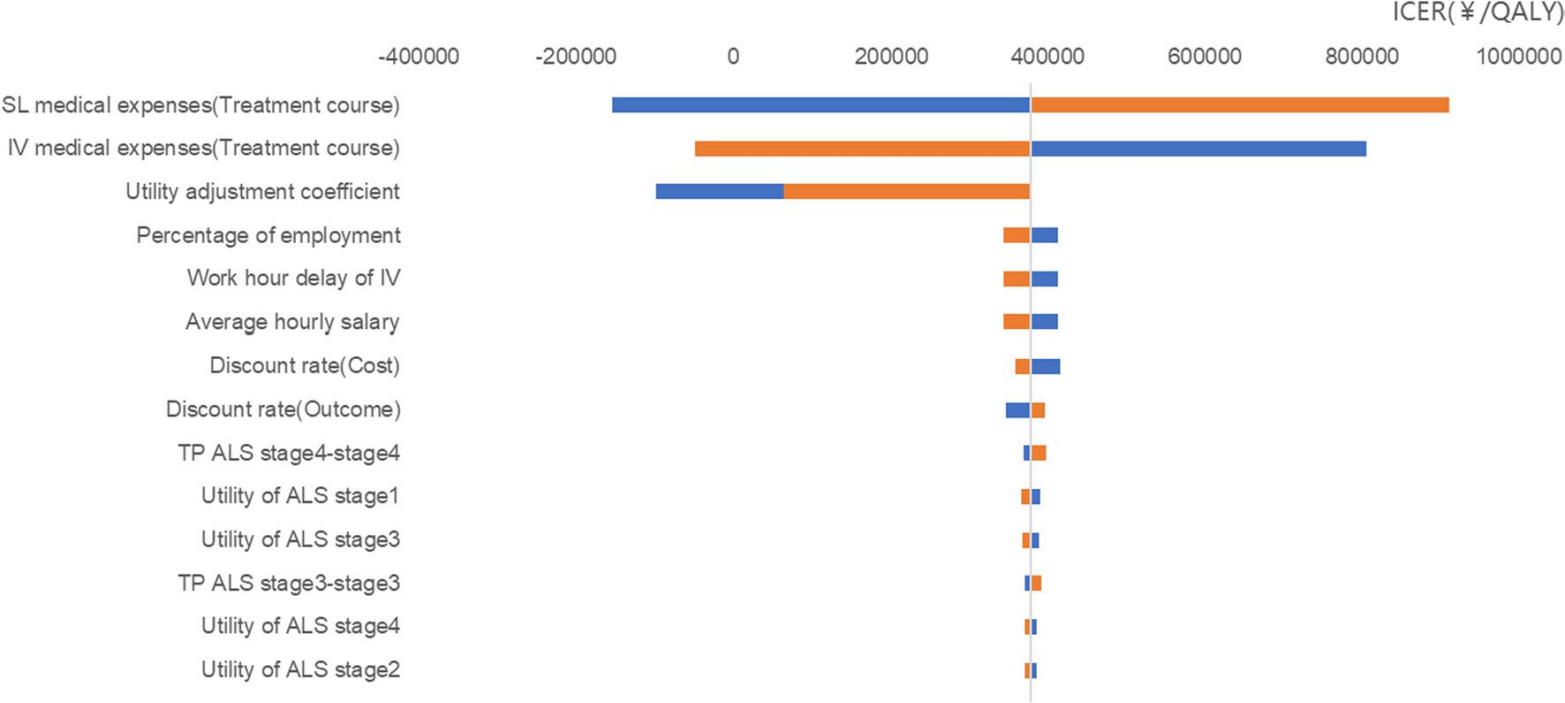
Cost-utility tornado chart. *ICER* Incremental cost effectiveness ratio, *QALY* Quality adjusted life year, *SL* Sublingual, *IV* Intravenous, *TP* Transition probability, *ALS* Amyotrophic lateral sclerosis

The one-way sensitivity analysis results showed that the cost parameters of both treatment groups and the SL utility adjustment coefficient were the most sensitive factors. The incidence rates of adverse events and associated costs were found to be insensitive factors. Refer to Fig. 2 for specific details.

**Fig. 3:**
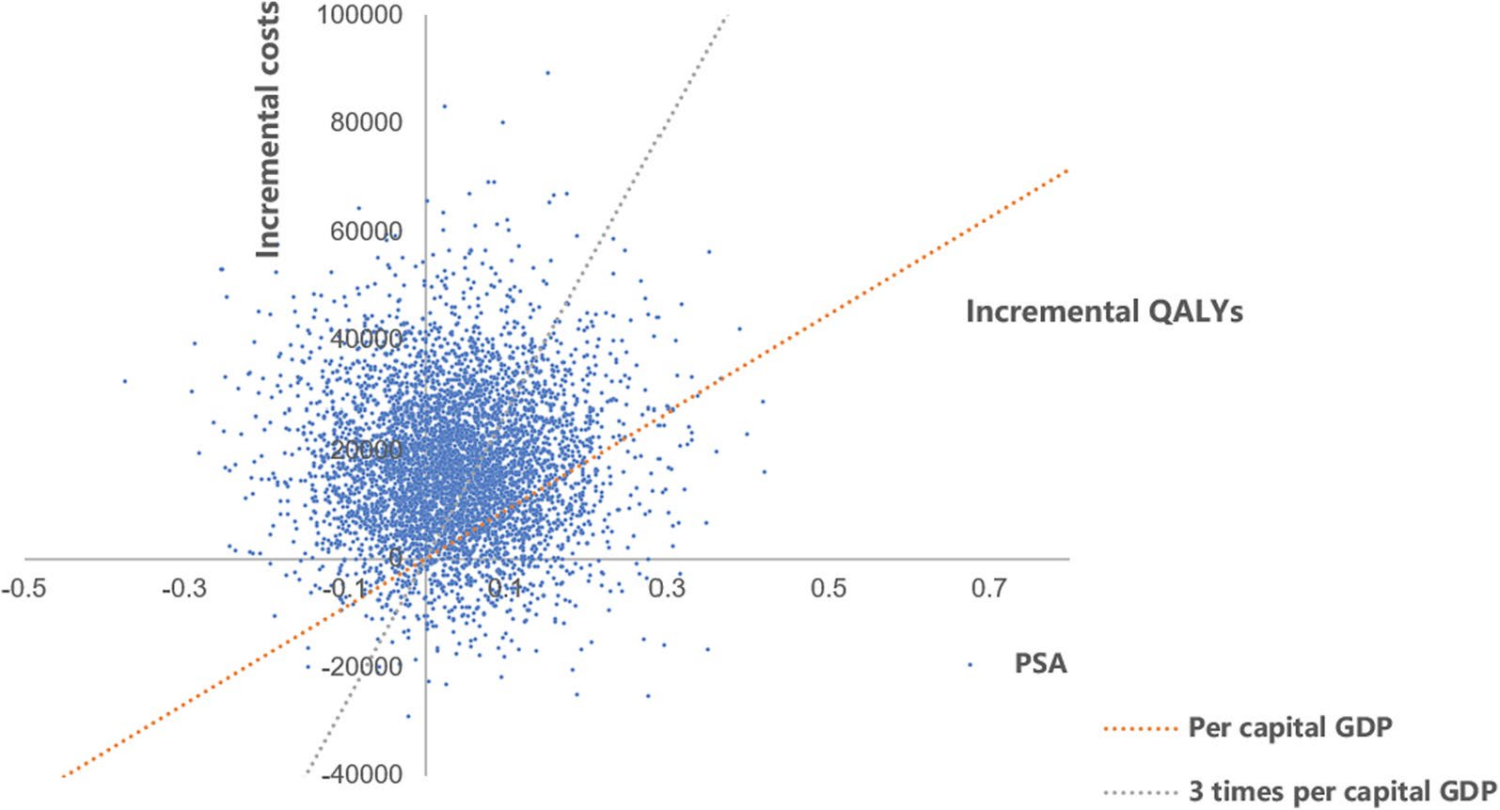
Cost-utility plane of SL versus IV edaravone. *QALYs* Quality adjusted life years, *SL* Sublingual, *IV* Intravenous, *PSA* Probabilistic sensitivity analysis

**Fig. 4:**
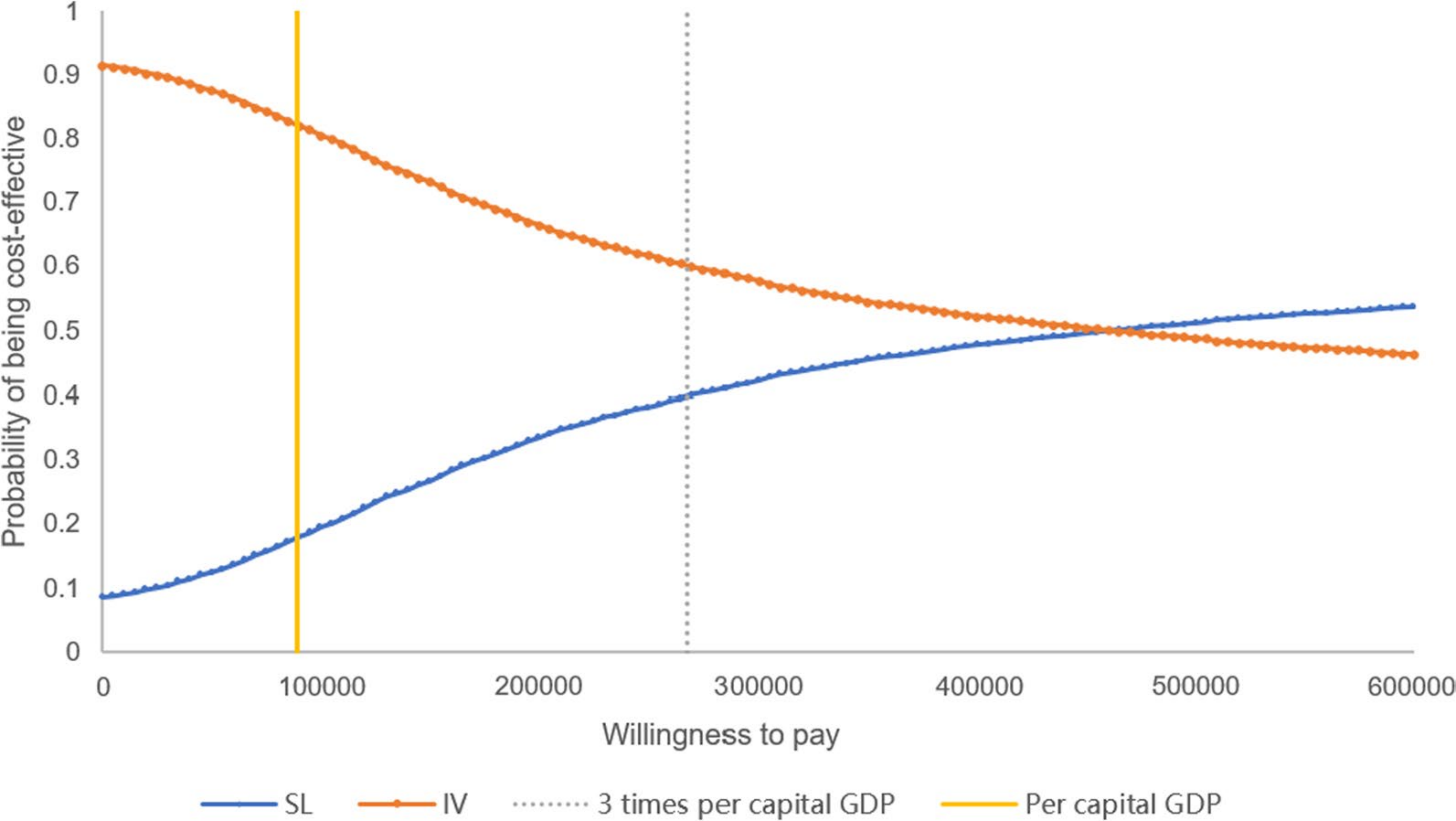
Cost-utility acceptability curve of SL versus IV edaravone. *SL* Sublingual, *IV* Intravenous

PSA results showed that the majority of the data points were located in the first quadrant in the cost-utility plane (Fig. 3), indicating higher costs and greater health benefits for the SL group in most scenarios. According to the cost-utility acceptability curve(Fig. 4), when the willingness to pay (WTP) per additional QALY is 3 times the per capita GDP of China, the probability of SL edaravone being cost-effective is 35.22%.

### Scenario analysis

Previous studies have shown significant variations in patient treatment experiences when using the same medication with different formulations, utility varies among outpatient, inpatient and home treatment [23]. In Ibrahim's study [24], it was demonstrated that there is a utility difference between children treated at home and those treated in the hospital, a utility adjustment coefficient of 1.147 was applied according to the study. In consideration of direct nonmedical costs, transportation expenses related to IV administration from and to the hospital was included. Patients with ALS, similar to children, require caregivers to accompany. Referred to Yuchen Zhou's study [25], the round-trip transportation cost for children and their accompanying guardians in the northern region of China was calculated to be ¥116.67, it was estimated to be average cost for ALS patients traveling to and from the hospital.

In the scenario of SL administered at home and IV administered in outpatient settings, the model calculations for a 20-year period showed that compared to IV edaravone, SL edaravone led to average cost savings of ¥26,664.94 and an additional gain of 0.234 QALYs, It indicated that the SL formulation was more cost-effective than the IV formulation.

## Discussion

ALS is a severe and highly fatal disease that imposes a substantial economic burden on patients, their families and society. Although ALS has a significant impact on patients' lives, it does not affect their intellectual capacity. Patients who receive timely and effective treatment to slow disease progression and extend their lives can still engage in cognitive activities. Therefore, the treatment of ALS has social significance. Edaravone has been proven to delay the progression of ALS in clinical practice. SL edaravone is an innovative formulation of edaravone that is bioequivalent to the IV form. The convenience of self-administering at home contributes to improving quality of life and reducing nonmedical costs such as transportation and home infusion services. Thus, it is worth promoting the clinical use of SL edaravone.

This study analyzed the costs and health outcomes of SL edaravone and IV edaravone by using a Markov model. The data were mainly extracted from the literature based on model assumptions. The study findings suggested that, in the context of home treatment, the medication cost for SL edaravone was greater than that for IV edaravone, but the costs related to work absence were lower, leading to slightly greater total costs. However, when considering the scenario of administering SL tablets at home versus IV in hospitals, SL edaravone resulted in overall lower costs. In terms of health outcomes, the SL group showed higher utility values, particularly when administering SL tablets at home versus IVs in the hospital, indicating better utility outcomes with SL tablets. Using 3 times the per capita GDP as the threshold for cost-effectiveness analysis, SL edaravone was found to not be cost-effective in the context of home treatment for both formulations. However, in the scenario of administering SL tablets at home versus IV at the hospital, the cost-effectiveness analysis demonstrated dominance, indicating the economic advantages of SL edaravone.

Sensitivity analysis revealed that the medication costs for both groups and the utility adjustment coefficient differences between the groups were critical variables which should be more concerned. The probability sensitivity analysis revealed a greater probability of higher costs and better outcomes in the SL group than in the IV group. Because the utility enhance of SL edaravone compare to IV edaravone was unclear, 95% CIs around the utility adjustment coefficient differences between the groups contains values less than 1, which resulted in plots falling on II and III quadrant, meant that there are chances that SL edaravone might be less effective than IV edaravone in model simulation. When the willingness-to-pay threshold was set at 3 times the per capita GDP, there were still probabilities of SL edaravone being cost-effective (around 35%).

Scenario analysis was based on the assumption of patients treated with same medication in inpatient, outpatient, and home treatment scenarios causes health utility difference. Gabriela's study [23] discussed subgroup analyses of the use of the same medication in inpatient, outpatient, and home treatment scenarios. The health utility values for these three scenarios were 0.200, 0.688, and 0.891, respectively. In China, home infusion services are rare, and therefore most patients need to visit hospitals for infusion treatments. SL tablet formulations allows patients to self-administer medication at home, thereby improving their quality of life and reducing the costs associated with transportation for medical visits. Scenario analysis results showed SL edaravone was cost-effective in the context of administering SL at home while IV in hospital. It indicated that treatment context can infect pharmacoeconomic evaluation results to some extent.

Limitations of this study include the following: (1) The study assumed that both groups of patients would adhere strictly to the medication regimen as outlined in the instructions. However, in reality, compliance with the SL tablet formulation might be greater, resulting in greater costs and better health outcomes. The potential differences in compliance between the two groups were not considered due to the lack of relevant data. (2) The analysis did not consider the work absence costs of caregivers per hospital visit. Nonetheless, this does not strongly affect the cost-utility evaluation results. Future clinical studies on SL and IV edaravone are suggested to provide further insights and references for economic evaluation.

## Conclusion

The innovation of SL edaravone is convenience of self-administering at home, thus contributing to quality of life enhance and nonmedical costs reduction. Using 3 times the GDP per capita of China in 2023 as the threshold, the SL edaravone was not cost-effective in the context of home treatment for both formulations. But was dominance to IV edaravone in hospital treatment. The results highlighted the importance of treatment context for health economic analysis.

## Data Availability

All data generated or analysed during this study are included in this published article.

## Abbreviations

ALS: Amyotrophic lateral sclerosis
SL: Sublingual
IV: Intravenous
ICER: Incremental cost-effectiveness ratio
QALYs: Quality of life years
CUA: Cost-utility analysis
PSA: Probabilistic sensitivity analysis
WTP: Willingness to pay
